# Characterizing Vaping Industry Political Influence and Mobilization on Facebook: Social Network Analysis

**DOI:** 10.2196/28069

**Published:** 2021-10-29

**Authors:** Michael Robert Haupt, Qing Xu, Joshua Yang, Mingxiang Cai, Tim K Mackey

**Affiliations:** 1 Department of Cognitive Science University of California, San Diego La Jolla, CA United States; 2 Global Health Policy and Data Institute San Diego, CA United States; 3 Department of Healthcare Research and Policy University of California, San Diego Extension La Jolla, CA United States; 4 S-3 Research San Diego, CA United States; 5 Department of Public Health California State University Fullerton, CA United States; 6 Global Health Program Department of Anthropology University of California, San Diego La Jolla, CA United States

**Keywords:** vaping, alternative tobacco industry, e-cigarettes, Facebook, social network analysis, social networks, ehealth, health policy

## Abstract

**Background:**

In response to recent policy efforts to regulate tobacco and vaping products, the vaping industry has been aggressive in mobilizing opposition by using a network of manufacturers, trade associations, and tobacco user communities, and by appealing to the general public. One strategy the alternative tobacco industry uses to mobilize political action is coordinating on social media platforms, such as the social networking site Facebook. However, few studies have specifically assessed how platforms such as Facebook are used to influence public sentiment and attitudes towards tobacco control policy.

**Objective:**

This study used social network analysis to examine how the alternative tobacco industry uses Facebook to mobilize online users to influence tobacco control policy outcomes with a focus on the state of California.

**Methods:**

Data were collected from local and national alternative tobacco Facebook groups that had affiliations with activities in the state of California. Network ties were constructed based on users’ reactions to posts (eg, “like” and “love”) and comments to characterize political mobilization networks.

**Results:**

Findings show that alternative tobacco industry employees were more likely to engage within these networks and that these employees were also more likely to be influential members (ie, be more active) in the network. Comparisons between subnetworks show that communication within the local alternative tobacco advocacy group network was less dense and more centralized in contrast to a national advocacy group that had overall higher levels of engagement among members. A timeline analysis found that a higher number of influential posts that disseminated widely across networks occurred during e-cigarette–related legislative events, suggesting strategic online engagement and increased mobilization of online activity for the purposes of influencing policy outcomes.

**Conclusions:**

Results from this study provide important insights into how tobacco industry–related advocacy groups leverage the Facebook platform to mobilize their online constituents in an effort to influence public perceptions and coordinate to defeat tobacco control efforts at the local, state, and federal level. Study results reveal one part of a vast network of socially enabled alternative tobacco industry actors and constituents that use Facebook as a mobilization point to support goals of the alternative tobacco industry.

## Introduction

Since the introduction of e-cigarettes—also known as electronic nicotine delivery systems (ENDS)—as a commercial product in the mid-2000s, their popularity has grown significantly. In 2018, 3.2% of US adults aged 18 years or older reported using e-cigarettes every day or some days [1]. Among middle and high school students in the US, e-cigarettes were the most commonly used tobacco product in 2019, with 5.4 million (20%) of youths reporting current use [2]. The global revenue from e-cigarettes in 2019 was US $15.7 billion, with a projected growth rate of 9.2% between 2020 and 2030 equating to an estimated US $39 billion in revenue by 2030 [3].This growth in sales is troubling from a public health standpoint, with evidence showing that e-cigarette usage is associated with breathing difficulty and cardiopulmonary health risks [4] as well as a higher likelihood to engage in risky behaviors among high school students [5]. In response to this growth in use and public health concerns, government agencies such as the US Food and Drug Administration have begun to assert their regulatory authority over e-cigarettes [6,7] along with a number of US states and municipalities that have instituted a variety of tobacco control policies (eg, sales bans of flavored tobacco products, tax regimes, license requirements, restrictions on youth access) to reduce the uptake, marketing, and sale of ENDS [8,9].

An abundance of evidence has shown that as tobacco control efforts expand, the tobacco industry has responded with a variety of messages and tactics to protect its business interests, including but not limited to lobbying, shaping the evidence base to support product use or harm reduction messages, policy substitution, and litigation [10-14]. As the overall reputation of the tobacco industry has declined over time, front groups and alliances with more reputable organizations have been especially important to its political activity because they shield such efforts from negative public perception about the industry [15-20]. Smokers’ rights groups were a particular group the tobacco industry used that attempted to mobilize smokers as a force to lobby policy makers to resist efforts that infringed on their “right to smoke” [17,21-23]. Although smokers’ rights groups were projected as being grassroots, they were a form of astroturfing, or “artificial grassroots campaigns created by public relations firms,” to increase the number of contacts that are made with policy makers in addition to traditional lobbying approaches [24].

The e-cigarette or “vaping” industry is also distinct in its structure compared to the overall tobacco industry. Specifically, the tobacco industry is highly concentrated among a few multinational corporations (China National Tobacco Corporation, Altria/Philip Morris International, British American Tobacco, Japan Tobacco International, and Imperial Brands make up approximately 82% of the global market share for cigarettes) [25]. These multinational tobacco actors also have a stake in the vaping industry as evidenced by the introduction by these companies of noncombustible products, mergers and acquisition activity with ENDS manufacturers, and other ownership of ENDS companies [26,27]. However, despite involvement from the tobacco industry, the ENDS industry has its own unique construction, including its own independent brands with national footprints (eg, Juul and the 35% share acquisition by Altria), smaller manufacturers and retailers of ENDS, and retailers independent of larger tobacco manufacturers [28]. Therefore, understanding the political resistance to expanding ENDS legislation can be informed by prior lessons learned from tobacco industry interference but will also need to evolve due to the differing landscape of actors associated with the growing ENDS industry.

A proactive leader in state-based policy efforts to regulate tobacco and ENDS is the state of California. Specifically, the state has been one of the leading jurisdictions in the United States in implementing local- and state-level tobacco control policies, including those related to smoke-free air and housing and tobacco prevention programs, which also includes measures directed at regulating ENDS [29,30]. For example, recent tobacco control legislation has included subjecting ENDS to existing antitobacco laws (Senate Bill [SB] X2-5), raising the purchasing age to 21 (SB X2-7), and imposing additional taxes on ENDS products (Proposition 56). In response, the vaping industry has been aggressive in mobilizing opposition to these policies by using a network of manufacturers, trade associations, and tobacco user communities, and by appealing to the general public in order to advocate for “vaper rights” [31-35]. One of the strategies used by the alternative tobacco industry has been to mobilize political action through social media platforms, such as the popular social networking site Facebook, which can extend the scope and reach of these antipolicy and advocacy efforts.

Previous studies examining the impact of social media platforms on the ENDS industry have identified user attitudes towards alternative tobacco products and behaviors of users [36-38]; characterized marketing tactics, sales strategies, and pricing of ENDS [39-46]; and identified geographic locations where people use ENDS [47]. However, few studies have specifically assessed how social media can influence antitobacco public policy and methods of social media mobilization among digital constituents [48,49]. This study builds on prior research by describing the membership and network structure of interest groups for alternative tobacco products on Facebook by using social network analysis (SNA). In order to observe mobilization across different online contexts, we conducted an exploratory investigation characterizing network structures of an alternative tobacco industry trade association Facebook page and a consumer-focused ENDS Facebook group.

## Methods

### Overview

This study used SNA to identify and characterize influential members and vape industry employees engaged with California chapters of 2 alternative tobacco interest groups. The proportion of influential members were compared between industry employees and nonemployees. An exponential random graph model (ERGM) was then used to detect statistically significant differences between vape employment status and likelihood to engage within the network, and a timeline analysis was used to investigate alignment with national- and state-level tobacco control policy events. All data collection and statistical and SNA analyses were completed in the computer programming languages Python (Python Software Foundation) and R (The R Foundation for Statistical Computing).

### Data Collection and Processing

This study used membership information available from 2 Facebook groups—the California Consumer Advocates for Smoke-Free Alternatives Association (CCASAA) and the Northern California Chapter of Smoke-Free Alternatives Trade Association (NC-SFATA)—to conduct SNA that characterizes the communication networks and identifies the position of influential members within and between these online communities.

The CASAA was founded in 2009 and describes itself as an advocacy group with an all-volunteer board and grassroots membership. It distinguishes itself as claiming to operate as a consumer organization and not a trade organization [50].The CASAA has a national Facebook page (address in New York) as well as pages for state chapters. The CCASAA Facebook page is for CASAA members residing in California and characterizes CASAA as “formed by people concerned about the continued availability of safer alternatives to tobacco” [51]. Members can “support CASAA’s goals by advocating in California for reasonable laws for products such as electronic cigarettes and smokeless tobacco products.” The CCASAA Facebook page has 195 members and is currently active.

The SFATA is a trade organization with a mission to “advocate for a reasonably regulated U.S. marketplace which allows our member companies to provide smoke-free products to adult consumers that are attractive in choice, while promoting a positive public image for vapor products…” [52]. Its membership includes “manufacturers, distributors, wholesalers, retailers, and the various service providers involved in their business” with membership opportunities open to the academic community and nonprofit organizations. The SFATA extends its national work into state and local level policy through its different state chapters. The NC-SFATA Facebook page characterizes SFATA as “representing the interests of the small- to mid-sized businesses by engaging with political decision makers, with advocacy at the national and state levels…SFATA is run and founded by the companies that built the vapor industry; with no ties or alliance to ‘Big Tobacco’” [53]. It discloses lobbying activity at the federal, state, and local levels as part of its range of services offered to members. The parent SFATA organization (address in Washington DC) operates an active Facebook page, and there are also numerous Facebook SFATA chapter pages in other states with varying levels of activity. The NC-SFATA Facebook page has 387 followers but has had limited activity since February 2017 (eg, only 2 recent posts in January 2021.) Despite its low recent activity, the page remains available and also provides an important digital record of how the group has mobilized to influence past California tobacco control legislation.

Data mining approaches using the Python programming language were used to collect publicly available data for all posts and comments from the Facebook pages of the 2 target groups over a 15-day period, from July 1, 2020, to July 14, 2020. This allowed us to collect all Facebook posts and comments retrospectively from users prior to July 1, 2020, and prospectively until July 14, 2020. Reposts were not removed as they were used for the purpose of analyzing interactions between posts and user interactions. Data were then restructured manually to a format suitable for conducting SNA. We note that public Facebook pages do not require request for joining or membership, and all posts, comments, and other information are publicly available to any online user.

Self-reported occupation data were also collected for public accounts and group members among these 2 target pages if published on a publicly available Facebook account profile. For users whose occupation data were unavailable on Facebook, additional searches on other online platforms with professional public profiles (eg, Twitter, LinkedIn) matched to public metadata profile information were conducted to ascertain possible work position status. These additional metadata were matched based on matching names with at least 1 other identifier before cross-referencing with the Facebook data. All data generated from occupational classification were in the public domain, and no individual identifiers of these accounts are reported in this study. Occupation data from users that did not have a link to their profile page in the metadata were marked as missing, as additional data could not be cross-referenced and matched.

### Social Network Analysis

SNA was conducted to detect influential members among alternative tobacco Facebook group communication networks reviewed. In the model presented in this paper, each node is a Facebook user associated with a California alternative tobacco trade association page (eg, CCASAA and NC-SFATA), and each edge (ie, link) between nodes represents reactions (eg, “like,” “love,” and “angry”) or comments on a post. As previous research has demonstrated the importance of distinguishing between active versus passive engagement with Facebook content [54-57], comments were weighted as twice the value of a reaction (eg, a like, emoji) in order to indicate higher engagement on posts. Generally, the source node is the user who produced the original post while the target nodes are users who reacted to or commented on a post. Within the context of this study, a node with a high in-degree centrality indicated that the user received a higher number of reactions on their posts while a high-outdegree centrality indicated users who were more active within their Facebook page.

Network visualizations for the individual CCASAA and NC-SFATA groups used a spherical layout, while the visualization that included both networks used the Fruchterman-Reingold layout algorithm [58] to emphasize differences in the communication structures between the 2 groups. Eigenvector centrality, which accounts for both the number of edges of each node and the level of connectivity of each node’s connections (ie, the extent to which a node’s connections is connected to others within the network), was used to measure influence within the network. Previous research has demonstrated that network structure measures such as Eigenvector centrality are reliable for detecting influential members within a network [59-61], and this measure has been validated in studies using data from both surveys and social media platforms [62-65]. Eigenvector scores were assigned to each node, with higher scores indicating higher influence within the network.

Proportions of users who were vaping industry employees were compared among the top 25% of users with the highest Eigenvector scores and remaining users with chi-square and a 2-proportion *z* test to test for statistical significance. Due to its increased usage within social network research [66] and previous work modeling social influence processes [67], a valued EGRM was used to detect a statistically significant influence between vape employee status and engagement within the network (ie, the likelihood of a tie formation) [68]. An ERGM is a statistical model that simulates alternative configurations of the observed network in order to determine the likelihood of a given structural feature, such as connections between nodes, which is referred to as “degrees” in SNA. Within the context of Facebook data used for this study, degrees correspond to the number of reactions and comments exchanged between users.

In order to model the value of the edges within the networks (as ERGMs traditionally only model binary ties), we employed an ERGM that accounts for valued edges by specifying a Poisson distribution as a reference for the distribution of edge values [69]. This study also examined homophily effects among ENDS industry–affiliated employees within the network. Homophily in social network research describes how members that share similar attributes, such as age, race, and gender, are more likely to interact with one another within a network [70] and has also been shown in health behaviors such as smoking [71]. Higher homophily among ENDS employees would indicate that users associated with the alternative tobacco industry are more likely to engage with one another on the Facebook group platform, suggesting higher coordination among members. The sum of reactions and comments for each post were calculated in order to identify influential posts based on whether the post was in the 75th percentile of engagement. Nodes with missing data for the ENDS employee status were kept in the analysis and imputed as not employed (46 nodes in total) in order to maintain network structure. This makes it possible that the models could be underestimating the strength of the effect that ENDS employee status and homophily have on the likelihood of communication ties.

### Policy Timeline Analysis

A timeline analysis was also conducted to detect associations between the number of influential posts and relevant federal and California state legislative events concerning e-cigarettes during the study period. This was done in order to observe how social media activity relates to policy outcomes, with histograms created to show the number of posts for each Facebook group by the month and year they were posted. Separate analyses for both groups were conducted with consideration to the different timeframes of activity between the organizations (the CCASSA activity was from February 2019 to June 2020, while NC-SFATA activity occurred between May 2015 and June 2017). Additionally, posts were categorized as either “Top 25% engagement” or “Remaining 75%” based on whether they were in the 75th percentile of posts that received the highest number of reactions and comments.

### Ethics

This study only involved the use of publicly available information in the public domain and did not include any interaction with social media users or other human participants. No personally identifiable information was included in the results of this study, and all results have been aggregated to prevent inadvertent disclosure of identifiable information. Hence, this study was not subject to ethics review.

## Results

### Comparing CCASAA and NF-SFATA Networks

A total of 292 active users (ie, nodes) among both CCASAA and NC-SFATA Facebook groups were included in the SNA from interactions consisting of posts, comments, and reactions resulting in a total of 509 edges (ie, connections between users). Of the total users, 246 had data linking to their public Facebook account and were also reviewed for identifying occupation data. Among these 246 users, 53 (21.5%) self-reported being employed by the alternative tobacco industry, which consisted of 10 retail workers (4.1%), 24 vape shop owners (9.8%), and 19 alternative tobacco industry public relations or marketing employees (7.7%). The predominant themes of Facebook posts and comments of these users included event invitations for activities directly mobilizing against tobacco control–related policies, public mobilization messages to encourage users to take individual action against tobacco control legislation, information about a tobacco control policy or introduced bill, and negative comments and opinions on tobacco control policies from the perspective of the alternative tobacco industry and ENDS users. A more in-depth qualitative description of these themes and specific content is being developed for separate analysis.

**Figure 1:**
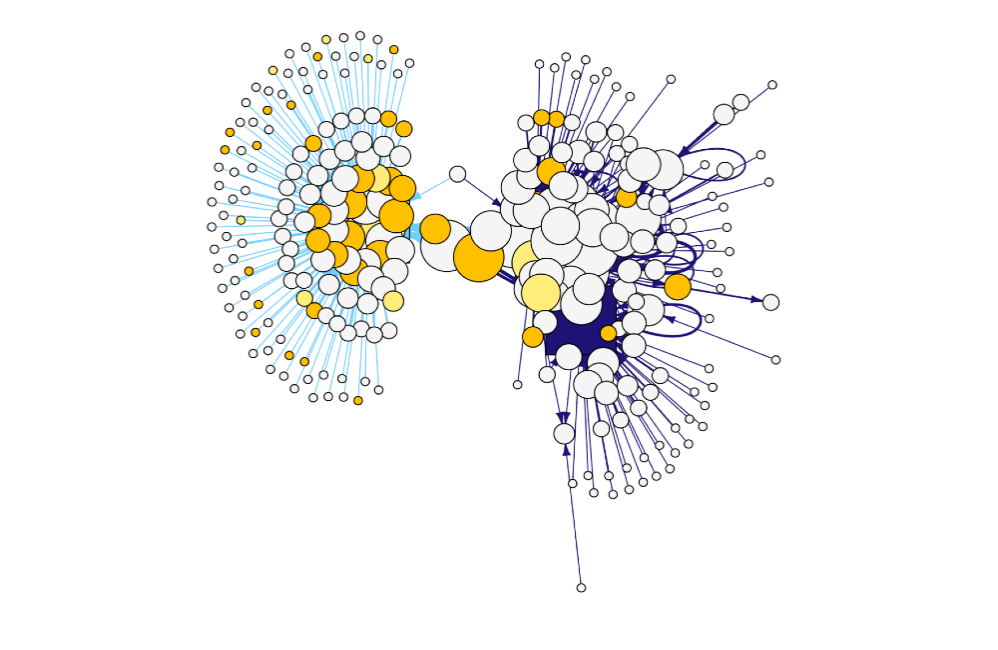
Consumer Advocates for Smoke-Free Alternatives Association and Northern California Chapter of Smoke-Free Alternatives Trade Association networks.

**Figure 2:**
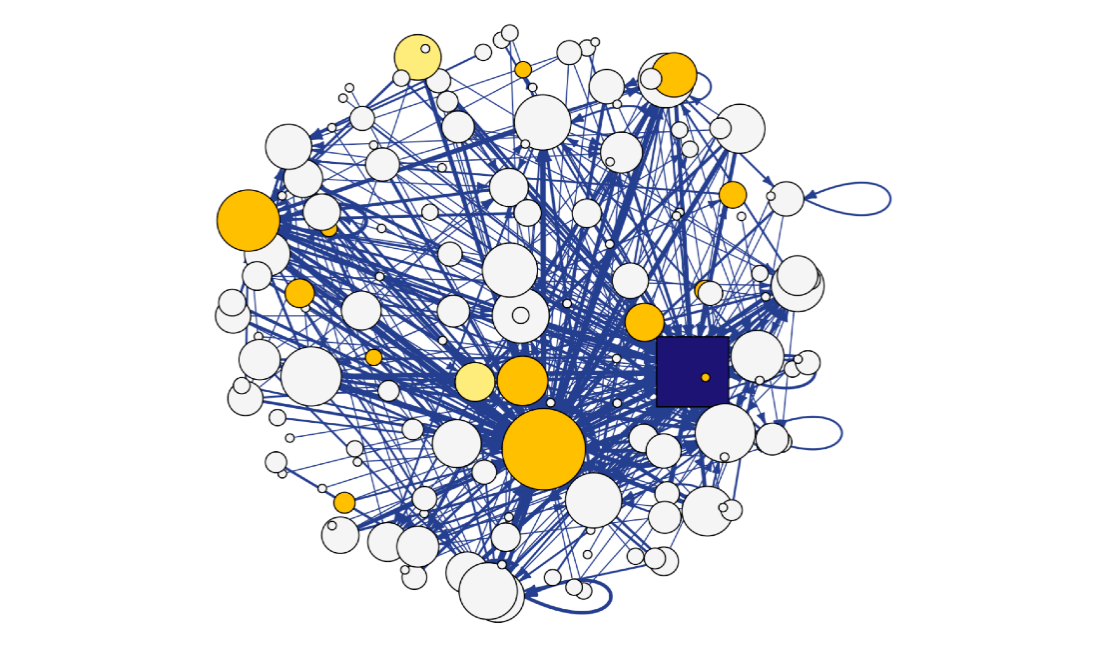
Consumer Advocates for Smoke-Free Alternatives Association network. The Consumer Advocates for Smoke-Free Alternatives Association is represented by the dark blue square.

**Figure 3:**
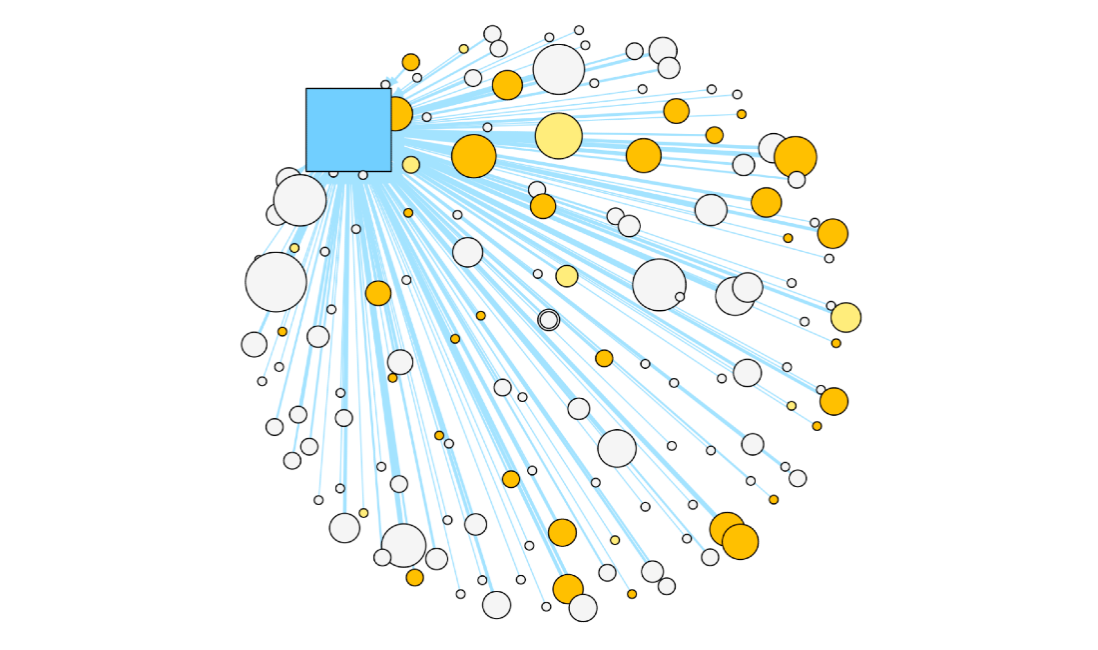
Northern California Chapter of Smoke-Free Alternatives Trade Association network. The Smoke-Free Alternatives Trade Association is represented by the light blue square.

SNA visualization of Facebook users in Figure 1 shows the overall network of both the CCASAA and NC-SFATA members while Figures 2 and 3 showcase how users are spread out among the Facebook pages. Light yellow nodes represent users who are alternative tobacco retail workers while dark yellow represents vape shop owners and alternative tobacco public relations or marketing employees. All remaining white nodes are nonalternative tobacco employee users. The source node is the user who produced the original post while the target nodes are users who reacted to or commented on a post. Edges representing online discourse on the CCASAA Facebook page are colored dark blue while activity on the NC-SFATA page is depicted by light blue edges. The size of nodes and edges depends on the weighted sum of the edge connections (ie, larger node and edge sizes equate to a higher number of reactions and comments).

Unsurprisingly, in an examination of the size of user nodes expressed in Figures 2 and 3*,* both the CCASAA and NC-SFATA are highly engaged within the networks as they are administrators of their respective Facebook pages. Most notably, the figures reveal that employees of the alternative tobacco industry are among some of the more engaged nodes. In Figure 1, both networks are shown to have mostly separate followings, with the CCASAA network having more engagement among nodes and the NC-SFATA network showing a more centralized, yet less engaged discourse. This is further supported when the degree distribution between networks is compared as shown in Table 1, where higher degree indicates more engagement. The mean degree for CCASSA users is 4.84 compared to 1.99 among NC-SFATA users, which shows that CCASAA users on average react, comment, or post more often. Additionally, CCASAA users have a higher degree across percentiles while NC-SFATA users consistently have a degree of 1 despite having a larger max value, which further indicates the centralized nature of the NC-SFATA communications. Network densities (ie, the ratio of the number of edges to the number of possible edges) were also calculated and show that the CCASAA network is denser (density 0.016) compared to that of the NC-SFATA (density 0.007), which is another indicator of greater activity levels among nodes within the CCASAA network.

Between the 2 groups, 8 Facebook users were identified as being active in both networks. Of these overlapping users, 6 were employed in the alternative tobacco industry, with 1 being a retail worker, 4 being vape shop owners, and 1 having served in a leadership role with the NC-SFATA. The involvement of a user with a leadership role in the SFATA chapter suggests the possibility of coordinated policy mobilization between these 2 California-based pro–alternative tobacco organizations and potential alignment of messaging and advocacy approaches. Additionally, Table 1 shows that alternative tobacco employees had a higher mean degree (mean 8.02) compared to the total network (mean 3.49), which indicates that these industry employees on average are more engaged within the groups compared to other users.

**Table 1:** Network statistics and degree distribution.

Statistics	Total network	CCASAA^a^ Only	NC-SFATA^b^ Only	Vape employee (CCASSA + SFATA)	Top 25% influential (CCASSA + SFATA)
Total nodes, n	292	148	152	53	76
Degree, mean (SD)	3.49 (11.52)	4.84 (10.28)	1.99 (12.17)	8.02 (25.18)	9.45 (21.43)
60th percentile (degree)	2	4	1	2	8
90th percentile (degree)	6	10	1	8	18
95th percentile (degree)	10	17	1	47	31
Max, n	151	83	151	151	151

^a^CCASAA: Consumer Advocates for Smoke-Free Alternatives Association.

^b^NC-SFATA: Northern California Chapter of Smoke-Free Alternatives Trade Association.

Table 2 shows that 32% (23/71) of the most influential members within the network were self-identified alternative tobacco employees compared to 17.1% (30/175) of other users, with this difference being statistically significant (*P*=.007). This indicates that alternative tobacco industry employees were more likely to be influential nodes within the communication network. Results from the chi-square test provide further evidence of an association between alternative tobacco industry employment and network influence, which shows that the distribution of alternative tobacco employees across influential and noninfluential members were statistically different from expected probabilities based on random chance (*P*=.002).

**Table 2:** Chi-square and 2-proportion z test comparing vape employee status between influential and noninfluential users (N=264^a^).

Tests for detecting statistical significance		Top 25% most influential	Remaining 75%
**Chi-square test^b^**			
	Expected probability	.25	.75
	Observed count (vape employees), n	23	30
**Two-proportion *z* test^c^**			
	Users, n	71	175
	Vape employees, n (%)	23 (32)	30 (17.1)

^a^Only users with links to Facebook profiles are included.

^b^*X^2^*_1_=9.566; *P* value=.002.

^c^*X^2^*_1_=6.078; *P* value=.007.

As shown in Table 3, the ERGM was run on the networks examined to detect statistically significant effects between being an alternative tobacco industry employee and the likelihood that a tie would form within the network, which in this study would signify engagement via a reaction or comment. The variable labeled “Nodefactor: *Vape*” measures how alternative tobacco employee status (coded as a categorical variable) influences the likelihood of a tie within the network. Model 2 additionally tests for homophily among alternative tobacco employees, labeled as “Nodematch: *Vape*.” The term “Nonzero” was included in the model to control for zero inflation of the network (ie, when a network is sparse but still has high interaction between nodes).

Results from Model 1 show that if a user was an alternative tobacco employee, then the log odds of sending a reaction or comment was 0.35 times greater than that of non–alternative tobacco employees when the density of the network is controlled for. This effect is statistically significant (*P*<.001) in both models. When homophily in Model 2 was tested for, there was a statistically significant negative effect (–0.27 log odds; *P*<.001) indicating that alternative tobacco users were less likely to engage with one another within the network. These findings indicate that while being an alternative tobacco employee increases the likelihood that a user engages within the CCASAA and NC-SFATA networks, these same users are more likely to interact with non–alternative tobacco industry employees within these online contexts.

**Table 3:** Exponential random graph model for valued edges to detect likelihood of being an alternative tobacco industry employee within the network.

Network parameter^a^	Model 1	*P* value	Model 2	*P* value
Sum (network density)	0.91	<.001	0.91	<.001
Nodefactor: Vape	0.35	<.001	0.42	<.001
Nonzero	–8.22	<.001	–8.08	<.001
Nodematch: Vape	—^b^	—	–0.27	<.001
AIC^c^	–163446	—	–163579	—

^a^Total nodes: 292; total edges: 500 (loop edges removed for the exponential random graph model); sample size per chain: 5000; thinning interval: 5000; reference distribution: Poisson.

^b^Not included in the model.

^c^AIC: Akaike information criterion. AIC within the context of an exponential random graph model measures deviance based on log-likelihood, which is calculated by summing the differences between predicted probabilities and observed values.

### Post Timeline Analysis

**Figure 4:**
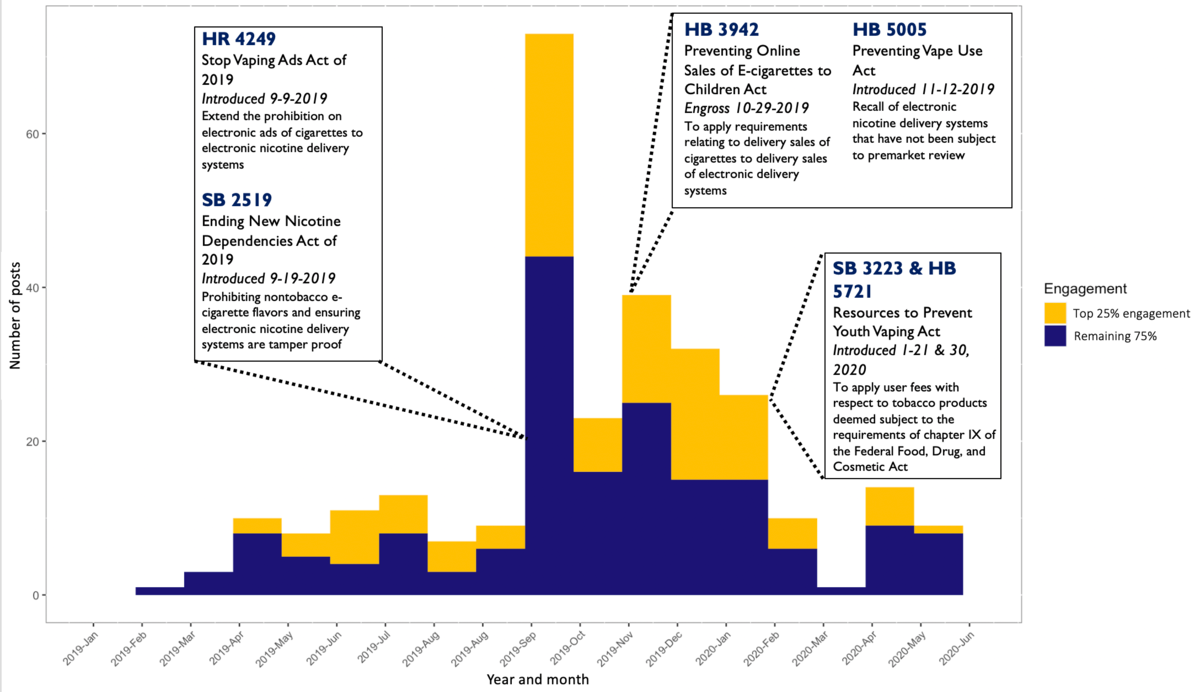
Consumer Advocates for Smoke-Free Alternatives Association posts timeline (2019-2020). HB: House Bill; HR: House of Representative; SB: Senate Bill.

**Figure 5:**
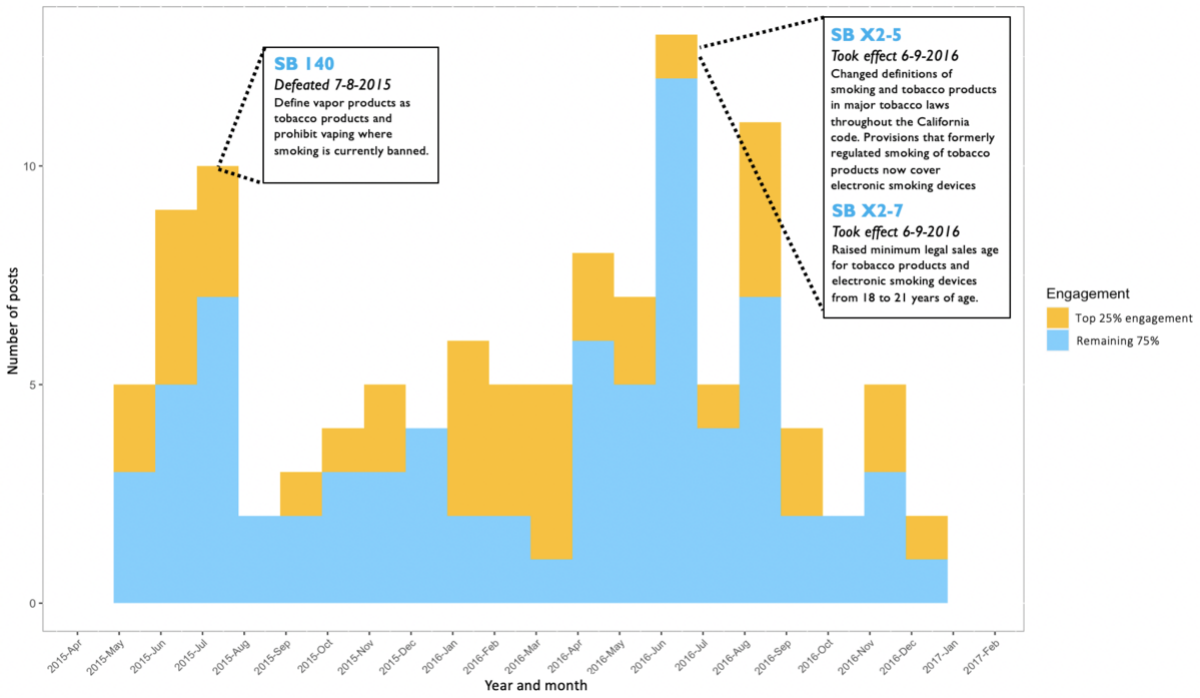
Northern California Chapter of Smoke-Free Alternatives Trade Association posts timeline (2015-2017). SB: Senate Bill.

Both Figures 4 and 5 show the post timelines for CCASSA and NC-SFATA content, respectively, with the proportion of posts receiving the top 25% most engagement filled in yellow. Each timeline includes markers detailing the date and descriptions of important vaping legislation during the time period in which posts were analyzed. Although not every post explicitly mentions a tobacco control policy, it can be interpreted that different forms of provaping messaging posted around legislative events can influence the opinions and mobilization actions of users. For example, an advertisement for a vaping device that shows up on a group member’s newsfeed still supports and communicates a provaping narrative whether or not it explicitly endorses a policy outcome. Users with higher exposure and engagement to pro–alternative tobacco messaging, which is the predominant theme within these Facebook groups, could be more likely to mobilize.

As seen in Figure 4, the largest number and largest proportion of highly engaged posts within the CCASAA network were created in September 2019, around the same time that federal legislation regulating alternative tobacco ads and calling for prohibiting of nontobacco e-cigarette flavors were introduced (House of Representative [HR] 4249 and SB 2519). Although posts had notably dropped in the following month in October 2019, there was a modest increase in activity in November around the introduction of additional regulation to the delivery sales of ENDS (House Bill [HB] 3942 and HB 5005). Importantly, this timeline generally depicts periods of increased activity and posts among the CCASAA Facebook users during periods when federal anti–tobacco and alternative tobacco legislation was introduced, both in the US Senate and House of Representatives.

Figure 5 shows a bimodal distribution of posts within the NC-SFATA group. The first spike of posts begins on May 2015 and peaks on July 2015 around the same time that the law SB 140 was defeated in the California Senate, which would have classified ENDS products as tobacco and extended the prohibition of tobacco smoking in public places to ENDS products. The second peak of posts happened during June 2016, when the same legislation succeeded in a subsequent legislative session and began to take effect as did other tobacco control legislation that increased the legal sales age of ENDS from 18 to 21 (SB X2-5 and SB X2-7). Despite having the highest number of posts within the timeline, the proportion of high engagement posts during June 2016 was low, possibly suffering from lower user engagement as anti–alternative tobacco legislation had already been successfully passed.

## Discussion

### Summary of Findings and Implications

Results from this study identify and characterize ways in which alternative tobacco interest groups act as virtual mobilization points across distinct online networks to influence tobacco control policy at both the federal and local level. This specifically includes our observation that alternative tobacco industry–affiliated actors are highly involved in antitobacco policy mobilization and advocacy activity with both an industry trade–focused group (NC-SFATA) that represents the interest of manufacturers, retailers, and distributors, but also a group that represents itself as a consumer organization for safer alternatives to tobacco. Specifically, our study found that there is a greater likelihood that alternative tobacco employees are within these Facebook user networks and that a higher proportion of industry employees are among the most influential members of the network. These findings are worrying as the presence of alternative tobacco employees and representatives may influence the factual narrative of tobacco and ENDS policy discussions due to financial interests and industry ties while not accurately conveying the concerns of consumers or consequences for public health outcomes.

Additionally, based on our timeline analysis, we observed that the highest number of messages with high engagement among these networks corresponded with dates tied to important tobacco control legislative events, evidencing increased activity around important policy decision-making windows. Some evidence of coordination between groups existed by examining network graphs with overlapping users in both organizations, and despite policy mobilization being active in both groups, they differed in their policy targets, with the CCASAA focusing on federal tobacco legislation and the NC-SFATA focusing on California state legislation. These results provide some indication of distinct mobilization and advocacy efforts at multiple policy-making levels. Although the purpose and messages of these activities clearly focused on disseminating information and influencing public perception for the purposes of defeating alternative tobacco legislation, levels of user engagement on these activities differed based on the group pages reviewed.

The network structure of the Facebook groups considered in this analysis differed in their density and post volume. The NC-SFATA Facebook group had lower density and more uneven distribution of engagement, while the NC-SFATA account was the main driver of posts to members. Additionally, the overall volume of posts was low compared to that of the CCASAA, and activity slowed after February 2017. The CCASAA had a denser network structure, a larger volume of posts compared to the NC-SFATA, and a more even distribution of activity among users that continues to the present. This suggests that the sustainability of engagement levels across interest groups is highly variable and may also be influenced by the presence and interaction of other affiliated groups (eg, SFATA and CASAA parent organizations that are also both active on Facebook).

Determining the specific factors that influence levels of engagement was beyond the scope of the current analysis; differences may be due to the more formal nature of trade associations (which may lend themselves to more linear communication structure) versus that of consumer-focused groups or to other organizational changes that occurred within the CCASAA. The existing literature, however, suggests that emotion plays a strong role in the alternative tobacco market as demonstrated in previous work which found business strategies used within the alternative tobacco market to be highly contested, volatile, and interwoven with competition, emotion, and conflicting beliefs [72]. The network characteristics shown in this study may indicate a higher emotional commitment among CCASAA members, a factor important in countering their political actions.

Although independent manufacturers and retailers within the alternative tobacco industry attempt to distinguish themselves from Big Tobacco and the goals of these 2 segments of the provaping movement may differ [28,73] (eg, implications of changes in tobacco use behavior and product sales due to dual use of cigarettes and e-cigarettes vs transition from cigarettes to e-cigarettes), they nevertheless are working to the same policy ends: to minimize restrictions on the marketing, sale, and use of tobacco and alternative tobacco products. As multinational tobacco companies engage in efforts to increase their share of the alternative tobacco market and expend large sums of money to lobby and fight ENDS regulations and restrictions, the less politically powerful and poorly resourced independent entities within the alternative tobacco industry may serve the role that front groups traditionally have served for the tobacco industry [74]. Through consumer groups and trade associations, they may attempt to influence policy makers through power in grassroots-based advocacy: mobilizing numerous “legitimate” voices from consumers and small businesses against tobacco and alternative tobacco control measures.

Further, results from our study likely only represent a very small segment of political influence exercised by the tobacco and alternative tobacco industry on social media platforms. For example, our study only examined 2 relatively small California-specific group pages and chapters; although the CASAA and SFATA’s parent organizations had much higher levels of activity and engagement on Facebook and Twitter (@CASAAmedia on has 33,000 and 22,000 followers on Facebook and Twitter, respectively, while @sfataorg has 15,000 and 10,000 followers on Facebook and Twitter, respectively), there are CASAA and SFATA Facebook group chapters in Florida, Missouri, Colorado, Connecticut, Hawaii, Texas, Arizona, and Ohio, to name a few other state-specific examples. There are also other pro–alternative tobacco industry groups engaged on social media that actively advocate against tobacco control measures that were not included in this study as we only focused on those associated with the state of California. Hence, although our study is limited to a single US jurisdiction and 2 pages, it is highly probable that lobbying and digital mobilization against tobacco control legislation via social media is occurring across multiple jurisdictions and a diversity of user groups, likely with similar ties to tobacco industry–affiliated employees and actors. This socially enabled online environment represents an important public constituency that can be activated against federal, state, and local alternative tobacco control measures, necessitating further research into ties with the broader tobacco industry and other front groups.

In response to this strategic use of Facebook (and other social media platforms) by the alternative tobacco industry to mobilize efforts to influence public perception and the outcome of federal and state tobacco control policy, public health stakeholders should expand their own efforts to mitigate and counter provaping narratives, particularly if they originate from alternative tobacco employees or lobbyists and misrepresent or include misinformation about current or pending tobacco control legislation. Public health stakeholders should also make a concerted effort to engage in these almost exclusively provaping virtual communities by establishing their own counter narratives highlighting the health and addictive harms of alternative tobacco products. Specifically, the posts on these pages appear to have an echo chamber effect, in that all the posts exclusively present a provaping narrative that is disseminated among users who are members of these pages. For Facebook pages that are open to the public, these discussions could spill over to other online communities, and in the absence of effective counter marketing, fact-checking, and health promotion, could lead to protobacco messages influencing opinions of other users. Platforms should also consider requiring disclosure of industry affiliations by page users or administrators and any corresponding potential conflicts of interest that may influence the type of information presented, including on individual posts that relate to claims about tobacco control policy.

### Limitations

As not every user was included in the *z* test comparing the proportion of alternative tobacco employees between influential and noninfluential users, it is possible that access to profile data could not be randomly distributed across alternative tobacco employee versus nonalternative tobacco users. If alternative tobacco employee status influences the likelihood of having available profile data, then this could have biased the results. Additionally, the results from this study only examined 2 California-affiliated organizations. More networks between local and national organizations would need to be analyzed in order to generalize findings about communication patterns in the overall alternative tobacco industry. As data were collected retrospectively, each network is also susceptible to data loss for posts collected before July 1, 2020. As mentioned in the methods section, the NC-SFATA is a Facebook page while the CCASSA is a Facebook community, which may account for many of the structural differences between communication networks. However, both formats share the same functions for interaction, so the main difference is between being listed as a group member versus only liking the page. Even if the format of the group on Facebook influences how users communicate with each other, it is possible that choosing a particular format could be done intentionally to better align with the communication goals of the page creator. Finally, the timeline analysis was conducted to clarify the association between the number of posts and legislative events and cannot be used to establish a causal connection.

### Conclusions

Future research should continue to characterize online communication strategies that differ between national and state or local alternative tobacco trade associations, consumer groups, and lobbying organizations in the social media sphere. This should include more in-depth characterization of formal coordination efforts on messaging, policy advocacy planning, and attempts to introduce misinformation about the impact of tobacco control legislation. For example, our study found that the NC-SFATA group had less engagement but a higher number of followers while the CCASSA group had fewer members but more engagement from these members. This may indicate specialization in the context of grass roots mobilization, advocacy, and policy substitution within the context of specific constituents, with SFATA focusing on mobilizing larger numbers of local and state actors and CCASSA focusing on smaller but more active engagement on federal issues. This potentially includes aligning with goals of each organization’s respective national parent associations, with the aim of defeating both federal and state legislation in a strategic and coordinated fashion. Unfortunately, the same coordination that is needed among federal regulators, public health professionals, and state health agencies to promote tobacco control policies may be absent from these social networking service platforms, meaning that pro–alternative tobacco narratives may unduly influence the policy-making process, threatening future tobacco legislation and implementation of policy already in place.

## Abbreviations

CCASAA: Consumer Advocates for Smoke-Free Alternatives Association
ENDS: electronic nicotine delivery systems
ERGM: exponential random graph model
HB: House Bill
HR: House of Representatives
NC-SFATA: Northern California Chapter of Smoke-Free Alternatives Trade Association
SB: Senate Bill
SNA: social network analysis
